# Mesenchymal Stem Cell-Cardiomyocyte Interactions under Defined Contact Modes on Laser-Patterned Biochips

**DOI:** 10.1371/journal.pone.0056554

**Published:** 2013-02-13

**Authors:** Zhen Ma, Huaxiao Yang, Honghai Liu, Meifeng Xu, Raymond B. Runyan, Carol A. Eisenberg, Roger R. Markwald, Thomas K. Borg, Bruce Z. Gao

**Affiliations:** 1 Department of Bioengineering, Clemson University, Clemson, South Carolina, United States of America; 2 Department of Pathology and Laboratory Medicine, University of Cincinnati Medical Center, Cincinnati, Ohio, United States of America; 3 Department of Cellular and Molecular Medicine, University of Arizona, Tucson, Arizona, United States of America; 4 New York Medical College/Westchester Medical Center Stem Cell Laboratory, New York Medical College, Valhalla, New York, United States of America; 5 Department of Regenerative Medicine and Cell Biology, Medical University of South Carolina, Charleston, South Carolina, United States of America; Centro Cardiologico Monzino, Italy

## Abstract

Understanding how stem cells interact with cardiomyocytes is crucial for cell-based therapies to restore the cardiomyocyte loss that occurs during myocardial infarction and other cardiac diseases. It has been thought that functional myocardial repair and regeneration could be regulated by stem cell-cardiomyocyte contact. However, because various contact modes (junction formation, cell fusion, partial cell fusion, and tunneling nanotube formation) occur randomly in a conventional coculture system, the particular regulation corresponding to a specific contact mode could not be analyzed. In this study, we used laser-patterned biochips to define cell-cell contact modes for systematic study of contact-mediated cellular interactions at the single-cell level. The results showed that the biochip design allows defined stem cell-cardiomyocyte contact-mode formation, which can be used to determine specific cellular interactions, including electrical coupling, mechanical coupling, and mitochondria transfer. The biochips will help us gain knowledge of contact-mediated interactions between stem cells and cardiomyocytes, which are fundamental for formulating a strategy to achieve stem cell-based cardiac tissue regeneration.

## Introduction

Cardiovascular disease, a pervasive clinical problem afflicting more than five million Americans, has an annual mortality rate of more than 20% [1]. The pathology of diseases, such as myocardial infarction, involves death of cardiomyocytes and leads to dysfunctional tissue. Transplantation of exogenous stem cells to the heart has been proposed to prevent or reverse heart failure [2]–[4]. It has been shown that not only can the transplanted stem cells transdifferentiate into cardiac phenotypes, they can also protect native cardiomyocytes. The protective effect from stem cell benefits the infarcted myocardium in a paracrine manner by secreting multiple soluble factors, which may act through reduction in infiltration of inflammatory neutrophils, inactivation of fibrogenic cells and scarring, stimulation of angiogenesis and vascularization, or recruitment and activation of resident cardiac stem cells [5], [6]. Besides the protective paracrine effect, contact-mediated intercellular interactions have been demonstrated to benefit myocardial repair and regeneration through three mechanisms that induce (1) cardiomyogenic differentiation of stem cells [7], [8]; (2) functional integration of the stem cells with host cardiomyocytes [9], [10]; and (3) delivery of molecules or even subcellular organelles from stem cells to enhance cardiomyocyte vitality and function [11]. However, a therapeutic procedure, which is heavily dependent on understanding signaling pathways and the structural and functional interactions between the transplanted stem cells and the host cardiomyocytes, has yet to be established.

The structural and functional integrations between cells are closely related to their particular contact-modes, including junction formation, tunneling nanotube connection, and cell fusion. In junction-formation mode, junctional proteins (e.g., connexins and cadherins) are distributed at the contact area between stem cells and cardiomyocytes: Connexins play an important role in electrical coupling, and cadherins do so for mechanical coupling [12]. Stem cells can also interact with cardiomyocytes by partial or full cell fusion process [13], [14]: Fused cells exhibit both stem cell and cardiomyocyte characteristics. A newly discovered mode of intercellular interaction between stem cells and cardiomyocytes is formation of thin-membrane channels (tunneling nanotubes). These nanotubular structures contain actin and microtubules to establish cytosolic connectivity and facilitate intercellular transmission of various cellular components [15]. Our knowledge of contact-mediated, in vitro stem cell-cardiomyocyte interactions is based mainly on conventional cell-culture models, which contain (1) an undefined number of contacting cells (one cell contacts multiple cells simultaneously); (2) an undefined population of contacting cells (homotypic and heterotypic cell contacts); and (3) undefined contact modes. These undefined cellular contacts make it difficult in conventional cell-culture models to interpret and recognize the functional interactions associated with one specific contact mode formed between stem cells and cardiomyocytes.

To address this issue, we designed laser-patterned biochips to allow only one contact mode to form between stem cells and cardiomyocytes to systematically study their intercellular interactions. In our biochips, two types of microenvironment (contact-promotive and contact-preventive microwells) were created by lithographic microfabrication methods. Individual stem cells and cardiomyocytes were laser-patterned into the microwells using the laser-guided cell micropatterning technique (LGCM) [16], which provides high spatiotemporal resolution for single-cell studies. Biochips such as these, with well-defined cell arrangements, can be used to form specific contact modes so that contact-mediated stem cell-cardiomyocyte interaction corresponding to each specific contact mode can be quantified systematically.

## Materials and Methods

### Ethics Statement

All experiments that involved animal use were performed in compliance with relevant laws and institutional guidelines. These experiments were approved by Clemson University's Institutional Animal Care and Use Committee through protocol AUP2010-032.

### Cell culture

Neonatal cardiomyocytes were isolated from three-day neonatal rats using a two-day protocol and cultured using high-glucose Dulbecco's Modified Eagle's Medium (DMEM) supplemented with 20% fetal bovine serum (FBS) and 1% penicillin streptomycin [17]. In previous characterizations, our isolation protocol yielded approximately 95% cardiomyocytes. Because of the size difference between cardiomyocytes and nonmyocyte cells, we were able to select and deposit only cardiomyocytes into the microwells during the laser-patterning procedure. Commercial rat mesenchymal stem cells (rMSCs) from bone marrow were purchased from ScienCell™ research laboratories and characterized by the company with antibodies to CD73, CD90, CD105, and oil red staining after adipocyte differentiation. The rMSCs were cultured using mesenchymal stem-cell medium (MSCM) from the same company and used for cell patterning before the fifth passage. The cardiomyocyte-culture medium was used as the coculture medium for the biochips during and after the laser-patterning procedure. Characterization of the rMSCs and cardiomyocytes used in our culturing system has been recently published [18]. To further characterize the rMSCs and cardiomyocytes in the microwells after laser-patterning procedure, MSCs were stained for CD90 and CD105 in contact-promotive microwells, whereas cardiomyocytes were stained for cardiac troponin T and myosin heavy chain in contact-preventive microwells (Figure S1). The geometric restriction within the microwells did not affect the expression of these cell-type specific markers.

### Biochip design and construction

The basic design concept was to create a microwell with defined geometrical restrictions that contained one rMSC and one cardiomyocyte. We produced two types of microwell: contact-promotive and contact-preventive. For each biochip, identical 9×9 microwells were created by attaching an elastomeric membrane with through holes onto a glass coverslip. The distance between two neighboring microwells was 200 µm. The microwell that promoted cellular contact was designed in a rectangular shape (50 µm long and 25 µm wide) that allowed the confined cell bodies (a single rMSC and a single cardiomyocyte) to form physical contact. The microwell that prevented cellular contact was designed in a dumbbell shape, with two circles (30 µm in diameter) connected by one channel (30 µm long and 15 µm wide). The curvature between the circular shape and the relatively narrow channel deterred formation of broad contact between a cell on one side of the channel and a cell on the other side. The microwell depth was 40 µm to restrict the cell bodies inside the microwells. Elastomeric membranes were microfabricated with polydimethylsiloxane (PDMS) using the standard procedure of photolithography and soft-lithography. The biochip assembly, treatment, and reuse are described in our previous publication [19]. Briefly, the biochips were cleaned, activated, sterilized by ethanol wash, oxygen plasma, and UV exposure, and coated with fibronectin before use. The biochips were placed inside the cell-deposition chambers, one biochip for each chamber, where individual rMSCs and cardiomyocytes were laser-patterned into the microwells.

### Laser-guided cell micropatterning (LGCM)

Consecutive placement of single rMSCs and cardiomyocytes into the microwells on the biochip is critical to this research. The high spatiotemporal resolution required in multiple-cell-type positioning was achieved using LGCM, which is thoroughly described in our previous publication [20]. After placing one biochip into the cell-deposition chamber, we used a nanopump system to inject nanoliters of cell suspension into the chamber above the biochip. An individual cell was selected and guided to the microwell at a speed of 150 µm/s horizontally and 50 µm/s vertically by the optical force generated from a weakly focused Gaussian laser beam (Movie S1).

**Figure 1:**
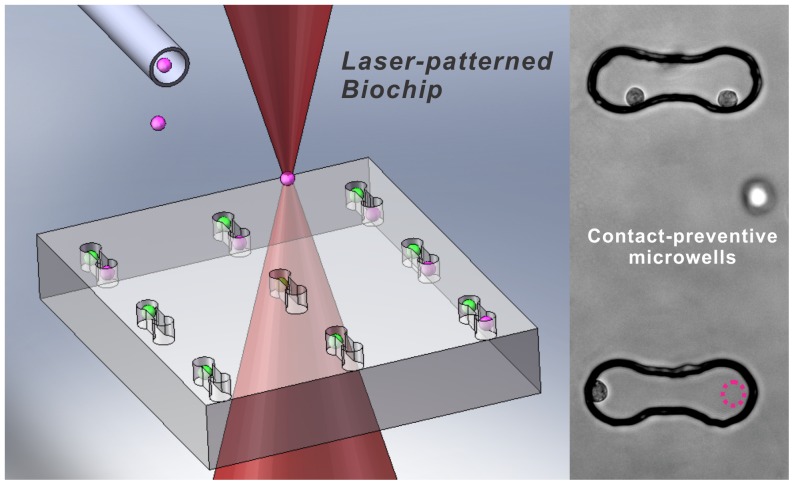
Schematic of the laser-patterning procedure. An rMSC is pumped out of the hollow fiber, trapped by the focused laser beam, and guided into the right side of a contact-preventive microwell (dotted pink circle) containing a cardiomyocyte (left side of the microwell). (pink: rMSCs; green: cardiomyocytes).

To create heterotypic cell pairs, cardiomyocytes were first laser-patterned into the left side of the microwells on the biochip, one in each microwell, and then rMSCs were patterned into the right side of the microwells, each of which already contained a cardiomyocyte, shown in Figure 1. The orientation of the microwell was determined by the triangular features established outside the central region, and thus cardiomyocytes were distinguished from rMSCs in the experiments that followed. Immediately after the laser-patterning procedure, the biochip was transferred from the cell-deposition chamber to a commercial 35 mm Petri dish containing cardiomyocyte culture medium. All biochips were incubated at 37°C in a humidified atmosphere of 95% air and 5% CO_2_, and the culture medium was changed every two days.

### Immunocytochemistry

On Day 5 (four days after laser-patterning), immunostaining was performed on the biochips. The cells were fixed, permeabilized, and blocked in 4% paraformaldehyde (10 min), 0.1% Triton X-100 (15 min), and 2% bovine serum albumin (BSA) with 4% donkey or goat serum. Next, the cells were labeled with the primary antibodies (mouse anti-sarcomericα-actinin (1∶400, Sigma-Aldrich), rabbit anti-connexin 43 (1∶200, Sigma-Aldrich), mouse anti-N-cadherin (1∶200, Sigma-Aldrich), and mouse anti-CD105 (1∶200, Santa Cruz Biotechnology Inc.)) at 4°C overnight. Excess primary antibodies were removed by a triple wash in PBS, and cells were stained with secondary antibodies (Alexa Fluor 488 donkey-anti-mouse IgG (1∶200, Invitrogen) and Oregon Green 488 goat-anti-rabbit (1∶200, Invitrogen)) at room temperature for 2 hrs. After three washes with PBS, the slides were prepared with ProLong antifade kit mounting medium (Invitrogen Inc.) including cell-nuclei-staining DAPI. The DAPI-labeled nuclei can be used to identify the cell position in the microwells. The nonspecific control groups (one group without primary antibodies and one group without secondary antibodies) were conducted simultaneously with the experimental groups. Immunostained cells were imaged using a confocal fluorescent microscope (Eclipse Ti, Nikon) equipped with a high-sensitivity quantitative monochrome camera (CoolSnap HQ2, Photometrics) and NIS-Elements imaging software (Nikon).

### Live cell-membrane and mitochondria labeling

In cell fusion studies, rMSCs and cardiomyocytes were labeled by DiI and DiO (Vybrant Multicolor Cell-Labeling Kit, Invitrogen), respectively, before the laser-patterning procedure. The serum culture medium was removed from the rMSC culture flask. The rMSCs were washed with warm PBS solution and then dissociated with 0.25% Trypsin-EDTA. The cells were collected in a 15 mL tube with serum culture medium and centrifuged into a pellet. The cells were resuspended by 2 mL serum-free medium (DMEM, 1% penicillin/streptomycin). Then, 10 µL DiI solution was added to the tube, and the cells were incubated in the dark at 37°C for 30 min. The cells were centrifuged into a pellet to remove the DiI solution and washed three times with serum-free medium. Then fresh serum culture medium was added to the tube for laser-patterning. Primary isolated cardiomyocytes were labeled with DiO using, except for the trypsin treatment, the protocol for rMSCs DiI-labeling.

To visualize mitochondria transfer from rMSCs to cardiomyocytes, the mitochondria of rMSCs were live-labeled by MitoTracker (MitoTracker Red CMXRos, Invitrogen). The lyophilized MitoTracker was dissolved in high-quality anhydrous dimethylsulfoxide (DMSO) to a final concentration of 1 mM as the stock solution. The labeling protocol was the same as that for cell-membrane labeling except for the use of a final MitoTracker concentration of 400 nM in serum-free medium.

### Statistical analysis

The occurrence of each contact mode was statistically analyzed according to our immunostaining results. First, each biochip was observed under bright field, and microwells containing round-shape cells, which were considered to be dead cells, were not used in the confocal examination. The percentage of occurrence of each contact mode was calculated by equation below (Eq. 1) for one biochip. Totally, 10 identical biochips were analyzed to collect the statistical results. Data were expressed as mean ± SD.
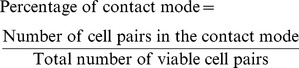
(1)


## Results

### Laser-patterned biochips

**Figure 2:**
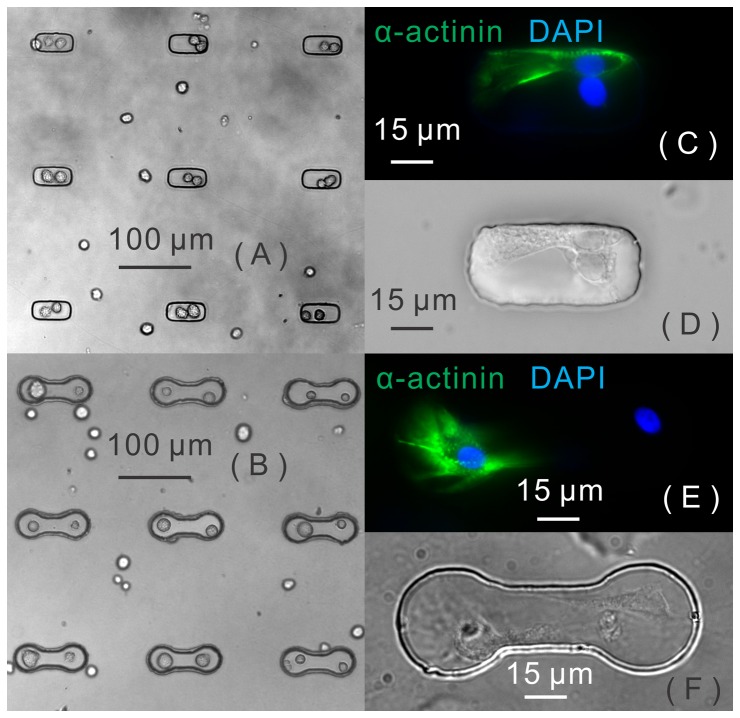
Laser-patterned biochips. Portions of laser-patterned biochips shown with (A) contact-promotive microwells and (B) contact-preventive microwells. After 48 hours culturing, the heterotypic cells (C, D) formed broad cellular contact in contact-promotive microwells; the heterotypic cells (E, F) did not form broad cellular contact in contact-preventive microwells. (α-actinin positive: cardiomyocytes, α-actinin negative: rMSCs, DAPI: nuclei).

The biochips with heterotypic cells were created by laser-patterning rMSCs and cardiomyocytes into the contact-promotive and -preventive microwells, shown in Figure 2 (A, B); the images were taken immediately after the laser-patterning procedure using a Zeiss phase-contrast microscope. The cardiomyocytes were on the left side of each microwell, and the rMSCs were on the right side. Typically, after being cultured 48 hours, the surviving cell pairs spread inside the fibronectin-coated microwells. Surviving heterotypic cell pairs were 58±3.1% of all laser-patterned cell pairs, slightly lower than that of conventional culture, which is consistent with the reports emphasizing the decreased viability on microwell-based single-cell analysis [21], [22]. In the following experiments, only the surviving cell pairs were studied. In Figure 2 (C, E), the DAPI-stained (blue) nuclei indicate the locations of the two cells inside one microwell, and the α-actinin staining (green) indicates cardiomyocyte structure. In contact-promotive biochips, 96±3.8% of the viable cell pairs formed broad contact between rMSCs and cardiomyocytes, as shown in Figure 2 (C, D); the remaining pairs, which did not contact each other (Figure S2 (A)), were not used in the statistical analysis. In contact-preventive biochips, cellular contact was impeded. Therefore, only 15±6.1% viable cell pairs were able to form broad cell contact by cell migration across the narrow channel. Further, 55±8.7% viable cell pairs did not form any type of cell contact as shown in Figure 2 (E, F), and the other cell pairs formed long-distance connections.

### Junction formation

**Figure 3:**
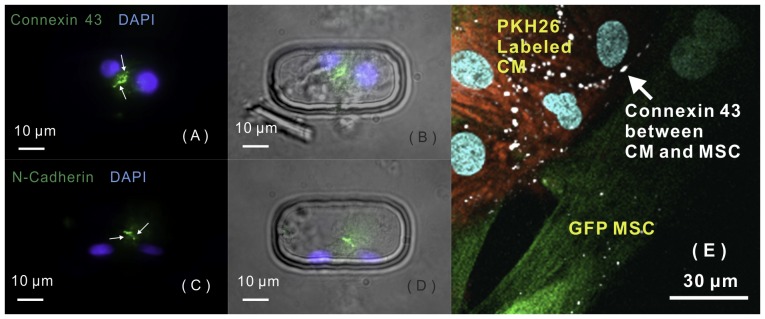
Junction formation between rMSCs and cardiomyocytes. Junctional distribution of (A, B) connexin 43 and (C, D) N-cadherin expressed at the contact area between rMSCs and cardiomyocytes in the contact-promotive microwells. (White arrows point to the junctional distribution of protein expression), (E) tetra-stained confocal image of cardiomyocyte (CM) and mesenchymal stem cell (MSC) coculture. The cell membranes of CM (red) and MSC (green) were labeled by different markers. Connexin43 was expressed at the junctional area between the CM and MSC membranes.

Junction formation between contacted cells was determined through imaging data on junctional protein expression: When the fluorescence image of junctional protein expression featured a thin line along the border of two cells in contact rather than a diffusive distribution, junction formation was counted. Two types of junctional proteins, connexin 43 and N-cadherin, were examined because of their importance to electrical and mechanical coupling between cardiac cells. For the heterotypic cell pairs, 84±3.9% were observed with connexin 43 expression, among which 28±6.5% exhibited junction formation, as shown in Figure 3 (A, B). The others exhibited only diffusive distribution throughout the cell bodies. In addition, 67±4.4% of cell pairs were observed with N-cadherin expression, among which 33±4.2% had junction formation, as shown in Figure 3 (C, D). The junctional-protein expression did not always cover the entire broad-contact area. In more than 90% of junction-formation mode, only a portion of the cellular contact area was found with junctional distribution of connexin 43 and N-cadherin. Figure 3E demonstrates the formation of connexin 43 between PKH26-labeled cardiomyocytes and a GFP-positive rMSC in our coculture dish. It is also important to note that the diffusely expressed junctional proteins inside the cytoplasm are not likely to support intercellular coupling; rather they represent newly produced, trafficking, or internalized connexin and cadherin molecules [12].

### Cell fusion

**Figure 4:**
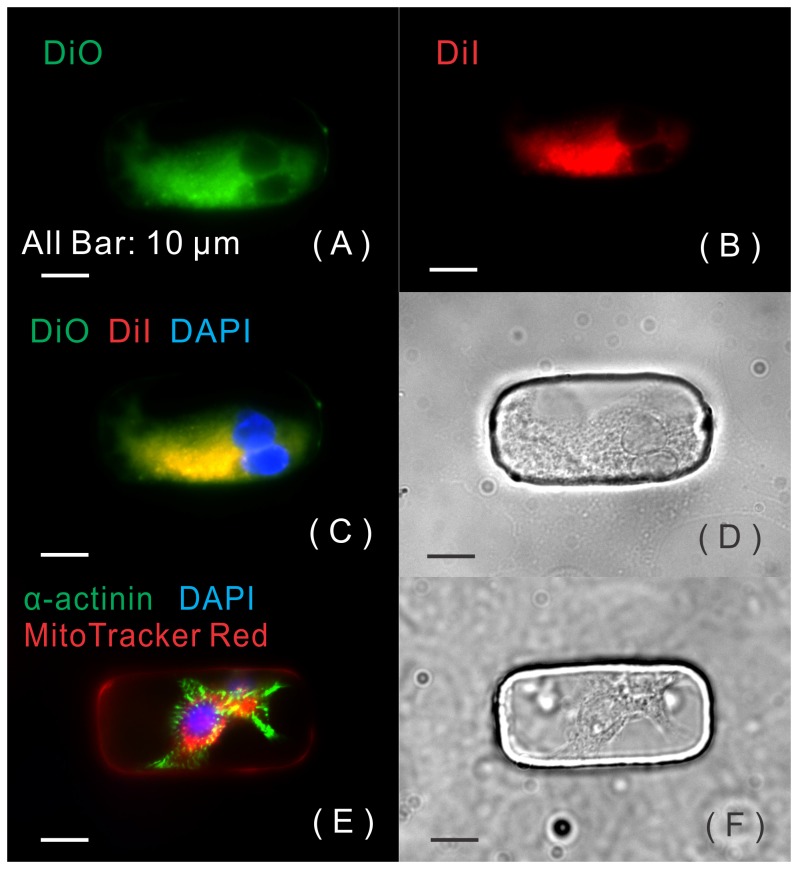
Cell fusion between rMSCs and cardiomyocytes. Full cell fusion (A–D) was observed by a DiO-labeled cardiomyocyte (A, green) fused with a DiI-labeled MSC (B, red) into double-nuclei, mixed labels (observed in fluorescent image, C), and membrane reorganization (observed in bright-field image, D). Partial cell fusion (E, F) was observed by rMSC mitochondrial transfer; labeled mitochondria (red) migrate across the membrane at contact area and accumulate near the cardiomyocyte nucleus.

In general, cell fusion is a natural process present from the time that a spermatozoid fuses with an ovule, and it is closely related to tissue formation, viral infection, and immune response [23]. The importance of cell fusion to regenerative medicine is increasingly recognized. For example, bone marrow-derived stem cells are able to fuse with mature somatic cells, such as Purkinje neurons, cardiomyocytes, and hepatocytes [24]. Between rMSCs and cardiomyocytes in contact, this phenomenon is an infrequently occurring form of intercellular interaction: Less than 1% occurrence was reported in previous coculture studies [25], [26]. To investigate the cell-fusion phenomenon at the single-cell level, we respectively labeled rMSCs and cardiomyocytes with DiI and DiO and laser-patterned them into the contact-promotive biochips. We found several double-nuclei cells with mixed labels (DiI and DiO), which indicated fused hybrid cells [11], as shown in Figure 4 (A–D). No clear contact area on the fused cell was observed in the bright-field image, which indicated full cell fusion accompanied by membrane reorganization. The ratio of cell fusion in our biochip-based coculture model varied from 0–6.3% among 15 contact-promotive biochips. Controlling cell contact in our biochips, we were able to evaluate the efficiency of cell fusion between rMSCs and cardiomyocytes at the single-cell level based on independent measurements of fluorescence mixing, nuclei centralization, and membrane reorganization.

In addition to full cell fusion, we observed partial cell fusion, in which two connected cells exchanged membrane and organelle components at their contact area [27]. In our research, mitochondrial transfer was used to identify the partial cell fusion within the contact-promotive biochips. The percentage of partial cell fusion was as high as 28±5.8% among contacted cell pairs in our biochips. We found that dye-tracked mitochondria from rMSCs migrated and accumulated in a contacted cardiomyocyte around its nucleus, as shown in Figure 4 (E). Unlike the membrane reorganization observed in the full cell fusion, the contact area between rMSCs and cardiomyocytes at their cell membranes was still identified, as shown in Figure 4 (F). The interacting cells maintained their stable cell-membrane structure, but fused in small areas, which resulted in direct cytoplasmic contact between the cells.

### Tunneling nanotube

**Figure 5:**
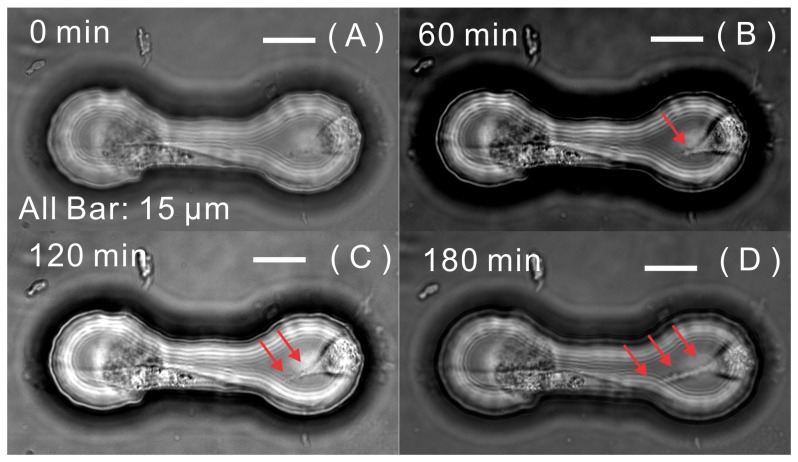
Tunneling nanotube extension from one rMSC. Time-lapse images (A–D) show the nanotube extension from an rMSC making contact with the cardiomyocyte in 180 minutes within the same microwell. (Red arrows point to the nanotube).

Long, thin connections have been characterized in cultured PC12 cells [28]. These tunneling, or membrane, nanotubes have been found to provide novel cell-to-cell communication, including organelle transfer [29] and electrical coupling [30], [31], over long distances. We used contact-preventive biochips to mimic the long-distance-communication condition; thus nanotube formation could be studied in an isolated model at the single-cell level. We observed that a beating cardiomyocyte in one contact-preventive microwell was connected by the nanotube extended from an rMSC (Movie S2). Time-lapse microscopy images, as shown in Figure 5 (A–D), demonstrated the process of nanotube formation in three hours: A nanotube extended from an rMSC to contact the cardiomyocyte in the microwell; it extended 20 µm in three hours.

**Figure 6:**
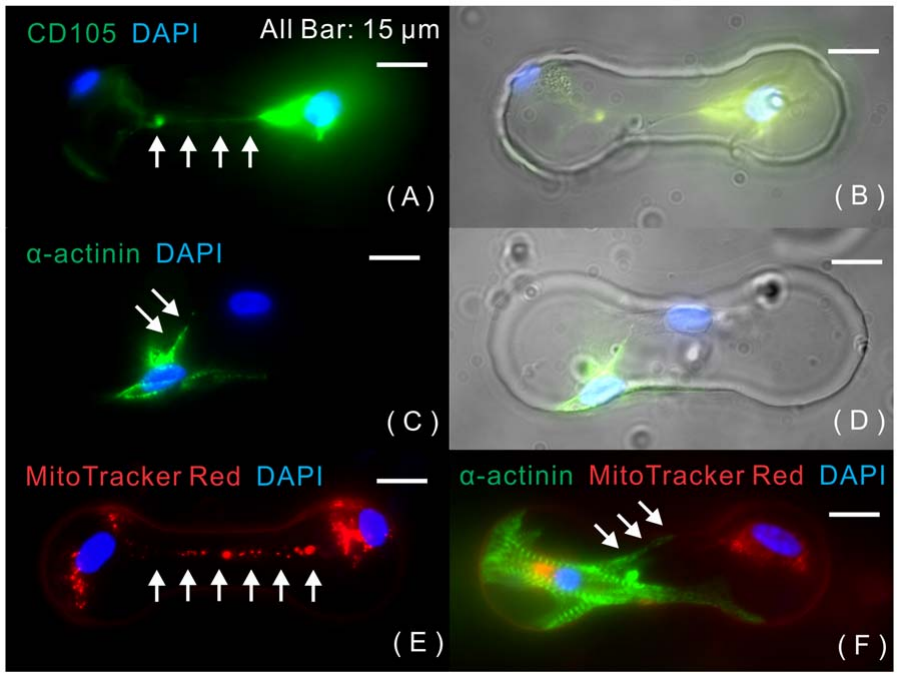
Long-distance communication between rMSCs and cardiomyocytes. Long distance connections between rMSCs and cardiomyocytes in contact-preventive microwells through either (A, B) rMSC-origin tunneling nanotube (determined by MSC surface marker, CD105) or (C, D) cardiomyocyte-origin filopodium-like structure (determined by cardiac marker, α-actinin). (E): Mitochondria transfer from one rMSC to its contacting cardiomyocyte. (F): Mitochondria transfer through the cardiomyocyte-origin filopodia and accumulate around the cardiomyocyte's nucleus. (White arrows point to the nanotubes).

The membrane protrusion extending from rMSCs was highlighted by staining rMSCs with specific membrane marker CD105, as shown in Figure 6 (A, B). In addition, the contacted cardiomyocyte was stained by α-actinin, as shown in Figure 6 (C, D). Our results showed that in a microwell, a tunneling nanotube developed from the membrane protrusion initiated from only rMSCs and extended towards the cardiomyocytes. Although some cardiomyocytes developed a filopodium-like structure that contacted distant rMSCs, its size was too large to be a nanotube. In the contact-preventive microwells, 18±4.1% of surviving, noncontacting heterotypic cell pairs were formed tunneling nanotubes from rMSCs, and 12±6.7% of cardiomyocytes exhibited a filopodia-like structure.

**Figure 7:**
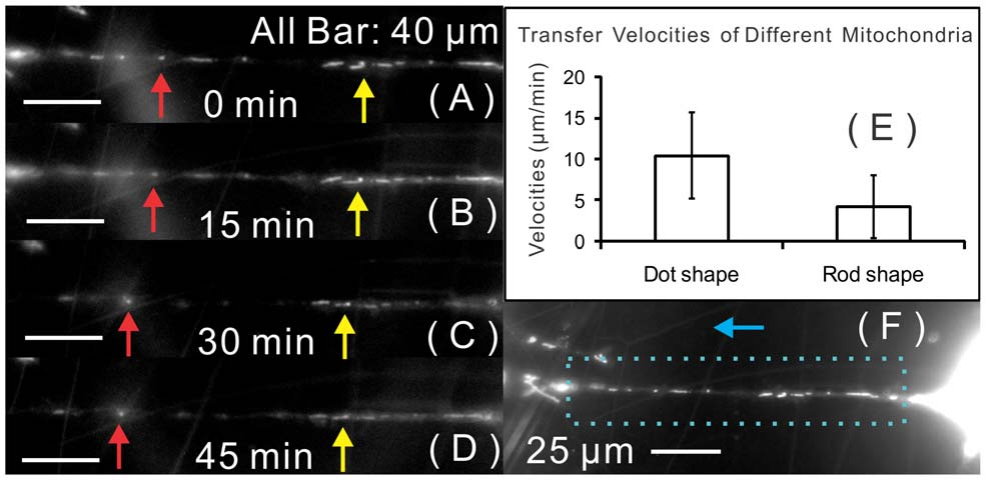
Mitochondria propagation through a nanotube. Time-lapse images (Zeiss Fluorescent Microscope, 63×, NA = 1.4) of mitochondrial propagation with a time interval of 15 minutes (A–D); red arrows point to a dot-shape (small) mitochondria, and yellow arrows point to a rod-shape (large) mitochondria. (E): The transfer velocities are highly dependent on mitochondrial size and shape; larger mitochondria have lower velocities. (F): rMSC and cardiomyocyte cell bodies connected by a nanotube, which is enlarged in the left column (A–D).

By tracking labeled mitochondria from rMSCs, we observed that mitochondria (red) were transferred from rMSCs to distant cardiomyocytes through tunneling nanotubes, as shown in Figure 6 (E). We also found that labeled rMSC mitochondria accumulated in the nucleus area of paired cardiomyocytes through filopodia-like extensions from the cardiomyocyte, as shown in Figure 6 (F). Although the total percentage of long-distance communication (including tunneling nanotubes and filopodia-like structures) was as high as 30% in contact-preventive biochips, the percentage of mitochondrial transfer was only 11±2.3% in contact-preventive biochips. Time-lapse microscopic recording was conducted to visualize the process of mitochondrial transfer, as shown in Figure 7 (A–D). The velocities of mitochondrial propagation along the tunneling nanotube were calculated by the digital image-correlation method. The transfer velocities were highly dependent on the size and shape of mitochondria (0.3–1 µm thick and up to 50 µm long [32]). The dot-shape mitochondria had higher transfer velocities than the rod-shape mitochondria, as shown in Figure 7(E).

## Discussion

Using our biochips, we isolated different contact modes between MSCs and cardiomyocytes at the single-cell level and investigated MSC-cardiomyocyte interactions through junctional coupling, cell fusion, partial cell fusion, and nanotubular connections. Although directly extrapolating the results to actual tissues is unfeasible, these data demonstrate that the single-cell models, each with a defined contact mode, are significant in providing basic interactional data at the molecular level, such as the direction of mitochondria transfer and the relationship between the molecular transfer rate and the extent of cell fusion. These data cannot be obtained systematically under a complicated cell-cell interactional environment. Our results suggest that 1) cell fusion, observed in microwell studies between stem cells and cardiomyocytes, may be an additional mechanism by which grafted cells can improve the infarcted myocardium; and 2) partial cell fusion and tunneling nanotubes can facilitate transfer of rMSC mitochondria to cardiomyocytes. Mitochondrial transfer from stem cells has been reported to rescue aerobic respiration in mammalian cells and may also promote cardiomyocyte reprogramming back to a progenitor-like state.

Among different culture models, means of cell fusion have varied widely, and therefore extensive cell fusion as an *in-vitro* artifact cannot be ruled out [33], [34]. Our concept of a defined microenvironment with single-cell resolution provides a tool to identify the cell-fusion phenomenon and quantitatively study its occurrence rate. Using a confocal fluorescence microscope, we found double-nuclei cells with mixed labels in contact-promotive biochips with a maximal percentage of 6.3%, which is higher than the average (0.7%) previously reported for cell fusion between stem cells and cardiomyocytes [26], [35]. Since the cell-fusion process requires that (at least) two interacting cells have a large contact area, there are two possible reasons for the high occurrence of cell fusion in our biochips: (1) Our 1∶1 rMSC-to-cardiomyocyte ratio was much higher than the seeding ratio of 1∶40 typical of conventional coculture [36]; (2) Our contact-promotive microenvironment increased the probability of broad cellular contact formation up to 95% in the limited space of a microwell. The cell-fusion phenomenon was also observed by patterning one rMSC and one cardiomyocyte in a larger circular microwell (Figure S2 (C, D)), but the occurrence decreased to 4.2%.

Partial cell fusion is characterized as fusion of the heterocellular plasma membranes and resulted in direct cytoplasmic contact and organelle transfer between the cells. In vivo partial cell fusion has been confirmed in the border zone of a rabbit myocardial infarction, where close contact between cardiac fibroblasts and dedifferentiated cardiomyocytes is accompanied by disruption of the basal lamina [27]. In our study, the partial cell fusion phenomenon was identified by mitochondrial transfer from rMSCs to contacted cardiomyocytes. Surprisingly, about 30% of heterotypic cell pairs with a broad contact area were found to have mitochondrial transfer; thus the occurrence of partial cell fusion was much higher than full cell fusion and approximately comparable to the rate of junction formation. This observation of partial cell fusion suggests that engrafted stem cells can contribute to the infarcted myocardium through partial cell fusion along with electrical and mechanical coupling through connexin 43 and N-cadherin. Partial cell fusion provides a unique opportunity for transfer of only cytoplasmic components without incorporation of the donor nucleus into the acceptor cells [37]. Although the benefit of partial cell fusion to neonatal cardiomyocytes has not been fully elucidated, it has been observed that rMSCs and adult-cardiomyocyte fused cells look like a type of cardiac progenitor: They demonstrate proliferative potential and express early cardiac transcriptional factors, but they lack contractile proteins [38]. Partial cell fusion opens a path for intercellular interactions to transport the cargo vesicle, mitochondria, growth factors and cytokines. It has been reported that MSCs synthesize and secrete a broad spectrum of growth factors, and cytokines such as VEGF, FGF, MCP-1, HGF, IGF-I, SDF-1, and thrombopoietin. Many of these factors have been demonstrated to produce beneficial effects on the heart, including neovascularization, attenuation of ventricular wall thinning and increased angiogenesis. Although partial cell fusion associated with mitochondrial transfer has been reported to facilitate cardiac reprogramming to progenitor-like cells, there might be other trafficking components that cross the fusion area that play key roles in the reprogramming procedure. It has been shown that a combination of three developmental transcription factors (i.e., Gata4, Mef2c, and Tbx5) rapidly and efficiently reprogram postnatal cardiac or dermal fibroblasts directly into differentiated cardiomyocyte-like cells [39], [40].

Tunneling nanotubes, a novel biological phenomenon in cell-to-cell communication over long distance, allow for transfer of cargo vesicles, mitochondria, and small molecules such as calcium [41], [42]. Actin filaments and microtubules are known to support the nanotubes between rMSCs and cardiomyocytes. In vivo nanotubular connection has been confirmed in mouse-heart tissue, in which fibroblasts extended membrane nanotubes between adjacent muscle bundles [43]. To study this long-distance communication phenomenon, we designed contact-preventive biochips to separate one rMSC and one cardiomyocyte inside two compartments connected by one narrow channel. In our study, the nanotubes were extended only from rMSCs. The other type of long-distance connection from cardiomyocytes resembled as filopodium-like structures protruding from the membrane. The overall amount of long-distance connections, including nanotubes and filopodia, was about 30% in our contact-preventive biochips, lower than that typically observed in conventional culture. A similar conclusion was made by Dr. Rustom's group, who found that nanotube formation among neuronal cells on a microstructured surface was 6% lower than on a conventional glass substrate [44].

We found that mitochondria from rMSCs can be transferred into cardiomyocyte cytoplasm either through rMSC-origin nanotubes or cardiomyocyte-origin filopodia. The mitochondria from rMSCs accumulated around the cardiomyocyte nucleus after transfer, which may modulate transcription-factor activities in cardiomyocytes through mitochondria-to-nucleus retrograde signaling. Recently, it has been reported that, after being transferred into cardiomyocytes, stem-cell mitochondria may cause somatic reprogramming [38]. In this report by Acquistapace and coworkers, although correlation between reprogramming and mitochondria transfer was confirmed using mitochondrial-depleted human MSCs, a genetic mechanism of reprogramming was not investigated. However, mitochondrial-retrograde signaling has been hypothesized to participate in the physiology and pathology of multiple cell types. For example, in skeletal myoblasts, mitochondrial stress caused upregulation of a number of genes involved in Ca^2+^ transport and storage, including Ryanodine Receptor I or II (RyR1 or RyR2), calreticulin, and calsequestrin [45]. Furthermore, mitochondrial-retrograde signaling has been hypothesized to induce phenotypic changes and progression in tumors. Increased expression of cathepsin L, a target gene involved in retrograde signaling, is an important factor in the invasive behavior of tumor cells [46]. Based on these facts, it could be hypothesized that, after being transferred into the cardiomyocytes, the MSC mitochondria would activate a mitochondrial-retrograde signaling pathway to induce dedifferentiation of the cardiomyocytes because of higher cytoplasmic Ca^2+^ concentration. According to the study on Zebrafish heart regeneration, it has been proposed that heart regeneration can be mediated by cardiomyocyte dedifferentiation and proliferation [47]. These findings suggest a regenerative pathway that is an alternative to well-accepted candidates, including differentiation, cell fusion, and paracrine effects. In particular, transfer of stem-cell mitochondria may explain why functional cardiac improvements are observed despite the fact that few of the donor cells are engrafted long-term.

In our contact-promotive biochips, connexin 43 and N-cadherin expression between two interacting cells was analyzed. The diffusive distribution of these two proteins was frequently observed in our biochips, but junctional distribution remained at one-third of the entire sample. Similar research has been conducted by Dr. Bursac's group [12], who found upregulation of expression of connexin 43 and N-cadherin in skeletal muscle cells and mesenchymal stem cells that were cocultured with cardiomyocytes. However, their results were obtained on only homotypic/heterotypic cell pairs with broad cellular contact. In the study we reported here, connexin 43 and N-cadherin were also expressed on noncontact rMSC-cardiomyocyte cell pairs in the contact-preventive biochips at a rate of approximately 50% but only in a diffusive manner (Figure S2 (B)). These results indicate that cellular contact not only upregulated protein expression, but also relocated expressed proteins to the contact area to facilitate cellular interaction through the junction mode. Although a diffusive distribution of connexin 43 and N-cadherin has been reported in the infarct zone [48] and in regions with implanted cells, further studies are necessary to investigate the precise distribution of junctional proteins at heterocellular interfaces within the heart.

## Conclusions

In our study, two types of biochips (contact-promotive and -preventive), which were created using laser-guided cell-micropatterning and microfabrication techniques, were used to study contact-mediated cellular interactions between rMSCs and cardiomyocytes. Four in vivo-relevant intercellular interaction modes, including junction formation, cell fusion, partial cell fusion, and tunneling nanotube, were modeled by our biochip systems. Investigation of these contact modes and functional integration of stem cells inside the heart may enhance our understanding of the mechanisms for cardiac-cell therapies.

## Supporting Information

Movie S1 **One cell was being trapped and guided during laser-patterning procedure.**
(MP4)

Movie S2 **One cardiomyocyte connected with one rMSC through tunneling nanotube was beating within a contact-preventive microwell.**
(MP4)

Figure S1 **The cells in the microwells were characterized to demonstrate that the geometric restriction within the microwells did not affect the expression of these cell-type specific markers.** The cardiomyocytes were characterized by (A, B) myosin heave chain and (C, D) cardiac troponin T, and rMSCs were characterized by (E, F) CD105 and (G, H) CD90.
(TIF)

Figure S2 **(A) noncontact cell pair in a contact-promotive microwell (α-actinin positive: cardiomyocytes, α-actinin negative: rMSCs, DAPI: nuclei), (B) diffusive connexin 43 distribution on noncontact cell pair in a contact-preventive microwell, (C, D) cell fusion in a circle-shape microwell with double-nuclei, mixed labels, and membrane reorganization.**
(TIF)
