# Supplementary figures and images for: Mesenchymal Stem Cell-Cardiomyocyte Interactions under Defined Contact Modes on Laser-Patterned Biochips
Source: PLoS One. 2013 Feb 13;8(2):e56554. doi: 10.1371/journal.pone.0056554 (PMC3572044; doi:10.1371/journal.pone.0056554)

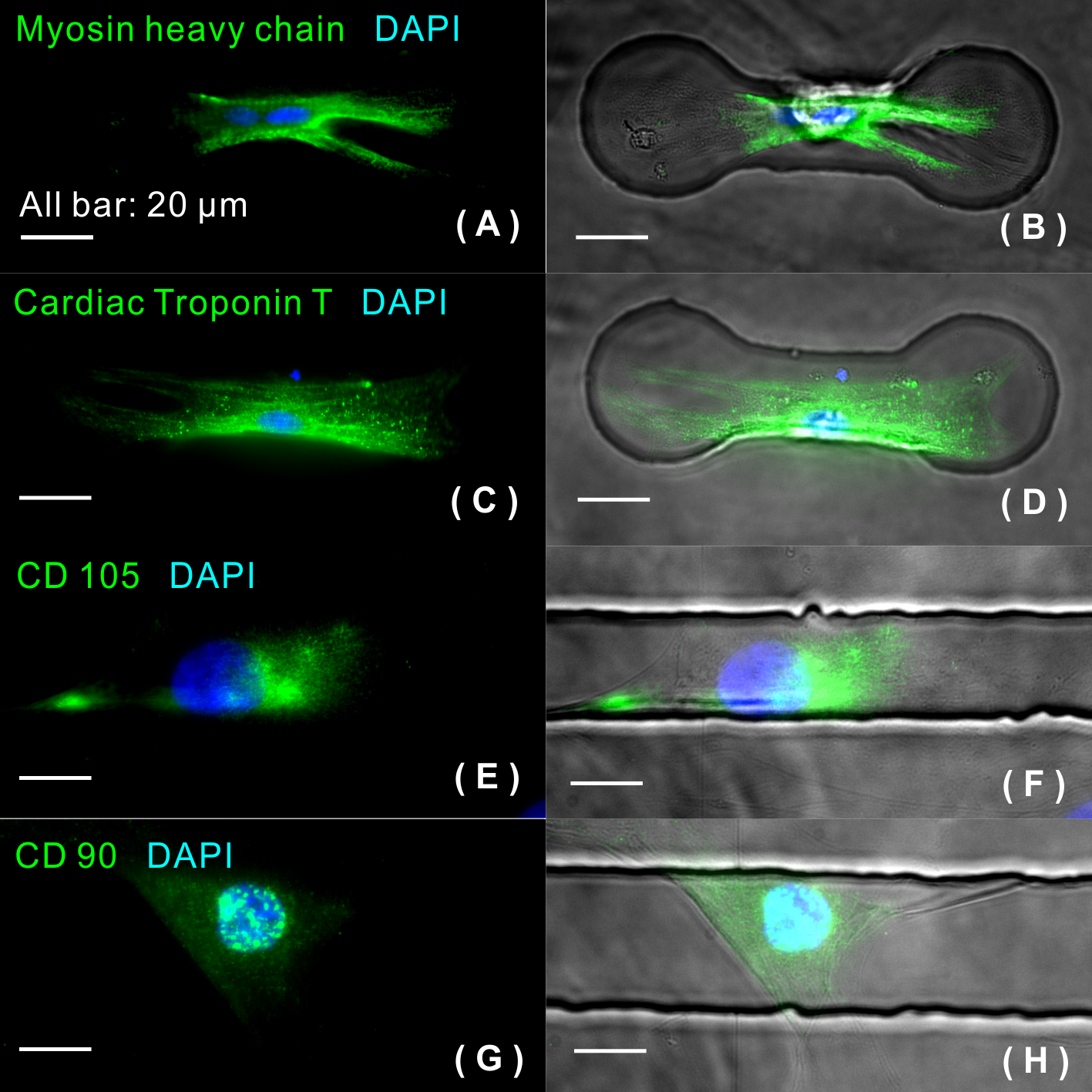

Supplement: Figure S1 — The cells in the microwells were characterized to demonstrate that the geometric restriction within the microwells did not affect the expression of these cell-type specific markers. The cardiomyocytes were characterized by (A, B) myosin heave chain and (C, D) cardiac troponin T, and rMSCs were characterized by (E, F) CD105 and (G, H) CD90. (TIF) [file pone.0056554.s003.tif]

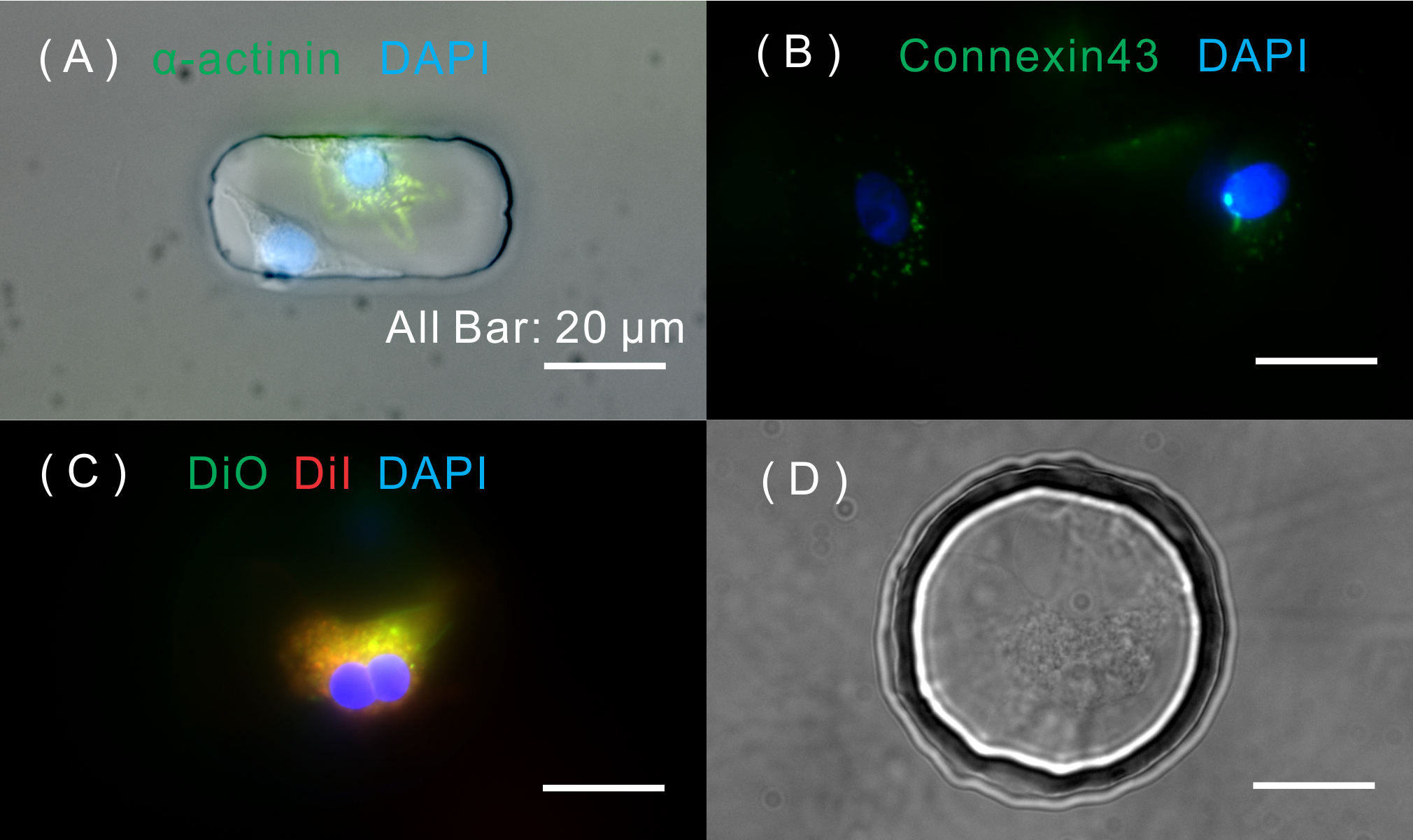

Supplement: Figure S2 — (A) noncontact cell pair in a contact-promotive microwell (α-actinin positive: cardiomyocytes, α-actinin negative: rMSCs, DAPI: nuclei), (B) diffusive connexin 43 distribution on noncontact cell pair in a contact-preventive microwell, (C, D) cell fusion in a circle-shape microwell with double-nuclei, mixed labels, and membrane reorganization. (TIF) [file pone.0056554.s004.tif]
